# Associations between total protein, globulin, and nasal Methicillin-Resistant *Staphylococcus aureus* (MRSA) colonization in US adults: results from the national health and nutrition examination survey 2001–2004

**DOI:** 10.3389/fimmu.2025.1585718

**Published:** 2025-05-30

**Authors:** Kanchao Chen, Xiaomeng Feng, Futong Liu, Yuping Fan, Tingting Zhang, Hui Wang, Sizhou Feng

**Affiliations:** ^1^ State Key Laboratory of Experimental Hematology, National Clinical Research Center for Blood Diseases, Haihe Laboratory of Cell Ecosystem, Institute of Hematology and Blood Diseases Hospital, Chinese Academy of Medical Sciences & Peking Union Medical College, Tianjin, China; ^2^ Tianjin Institutes of Health Science, Tianjin, China; ^3^ Haihe Laboratory of Cell Ecosystem, Tianjin Medical University, Tianjin, China; ^4^ Department of Hematology, The Affiliated Yantai Yuhuangding Hospital of Qingdao University, Yantai, China

**Keywords:** MRSA colonization, total protein, globulin, PSM, NHANES

## Abstract

**Objective:**

There is limited evidence on the association between total serum protein (TP), serum globulin (GLB), and Methicillin-Resistant *Staphylococcus aureus* (MRSA) nasal colonization. The purpose of this study was to investigate the association between TP, GLB, and MRSA nasal colonization in US adults with data derived from the National Health and Nutrition Examination Survey (NHANES).

**Methods:**

Using NHANES 2001–2004 data, we employed propensity score matching (PSM) to control confounders, weighted logistic regression to evaluate associations of TP and GLB with MRSA colonization, restricted cubic splines (RCS) for non-linear analysis, and subgroup and sensitivity analyses for validation.

**Results:**

Among 7,585 adults, 1.31% (n = 99) had MRSA nasal colonization. Adjusted multivariable regression identified TP and GLB as independent protective factors (TP: OR=0.92, 95%CI 0.88–0.96; GLB: OR=0.91, 95%CI 0.86–0.97; p< 0.05 for all). Categorizing TP and GLB into quartiles (Q4 vs. Q1) reinforced this association (TP: OR=0.21, 95%CI 0.07–0.59; GLB: OR=0.28, 95%CI 0.12–0.67; p< 0.05 for all) with consistent results post-PSM. Restricted cubic splines confirmed dose-dependent negative correlations. Subgroup analyses and sensitivity analyses supported the robustness of these findings.

**Conclusion:**

There was a negative correlation between TP, GLB, and MRSA nasal colonization in participants aged 18 years or older. Our data support the protective role of TP and GLB in MRSA colonization, and the specific mechanisms of these biomarkers in MRSA colonization and their clinical implications require further investigation.

## Introduction

*Staphylococcus aureus* (SA) asymptomatically colonizes the skin, mucous membranes (e.g., nares), and intestines in 20%–30% of individuals, serving as a reservoir for invasive infections (1, 2). This colonization significantly increases the risk of infection by providing a reservoir for pathogens, which in turn leads to severe complication rates and mortality. Methicillin-Resistant *Staphylococcus aureus* (MRSA) infections lead to prolonged hospital stays and associated increased healthcare costs (3–7). Globally, MRSA-related attributable deaths surged by 127% between 1990 and 2021, with the most pronounced increases among adults aged ≥70 years (over 80% mortality rise), despite declining trends in children under 5 years (8). In the United States alone, MRSA remains a leading cause of bacteremia, responsible for approximately 100,000 serious infections and 20,000 deaths annually (9). Prevention of MRSA colonization and infection has become critical as effective treatment options are diminishing (10).

The risk of MRSA colonization and infection is strongly related to immune function and nutritional status (11). Immunosuppressed or immunocompromised patients, such as blood autologous graft recipients, long-term immunosuppressed patients, and long-term hospitalized elderly individuals, have a significantly higher risk of colonization by MRSA and other multidrug-resistant organisms (MDROs), including carbapenem-resistant Gram-negative bacteria (MDR-GNB) and vancomycin-resistant enterococci (VRE) (11–18). However, most studies have focused only on the relationship between total serum protein (TP) and serum albumin (ALB), which are associated with immune function and nutritional status, and MRSA colonization, and there is a lack of studies that have simultaneously assessed the relationship between TP, serum globulin (GLB), and MRSA colonization. The purpose of this study was to investigate TP and GLB in a general population-based sample of U.S. residents to examine their association with concurrent MRSA nasal colonization.

## Method

### Study design and data source

NHANES is a nationwide research administered by the Centers for Disease Control and Prevention (CDC) to evaluate the health and nutritional status of both children and adults in the United States (19). NHANES employed a complex multi-stage probability sampling design to select its participants. Nasal colonization data for SA and MRSA are only available for the period 2001-2004 and we only included records of nasal Staphylococcus aureus colonization in U.S. adults older than 18 years of age. We therefore merged the datasets from the 2001–2002 and 2003–2004 survey periods for the purpose of analysis. NHANES was approved by the National Center for Health Statistics Research Ethics Review Board, and all participants provided written informed consent (20).

### Study population

**Figure 1:**
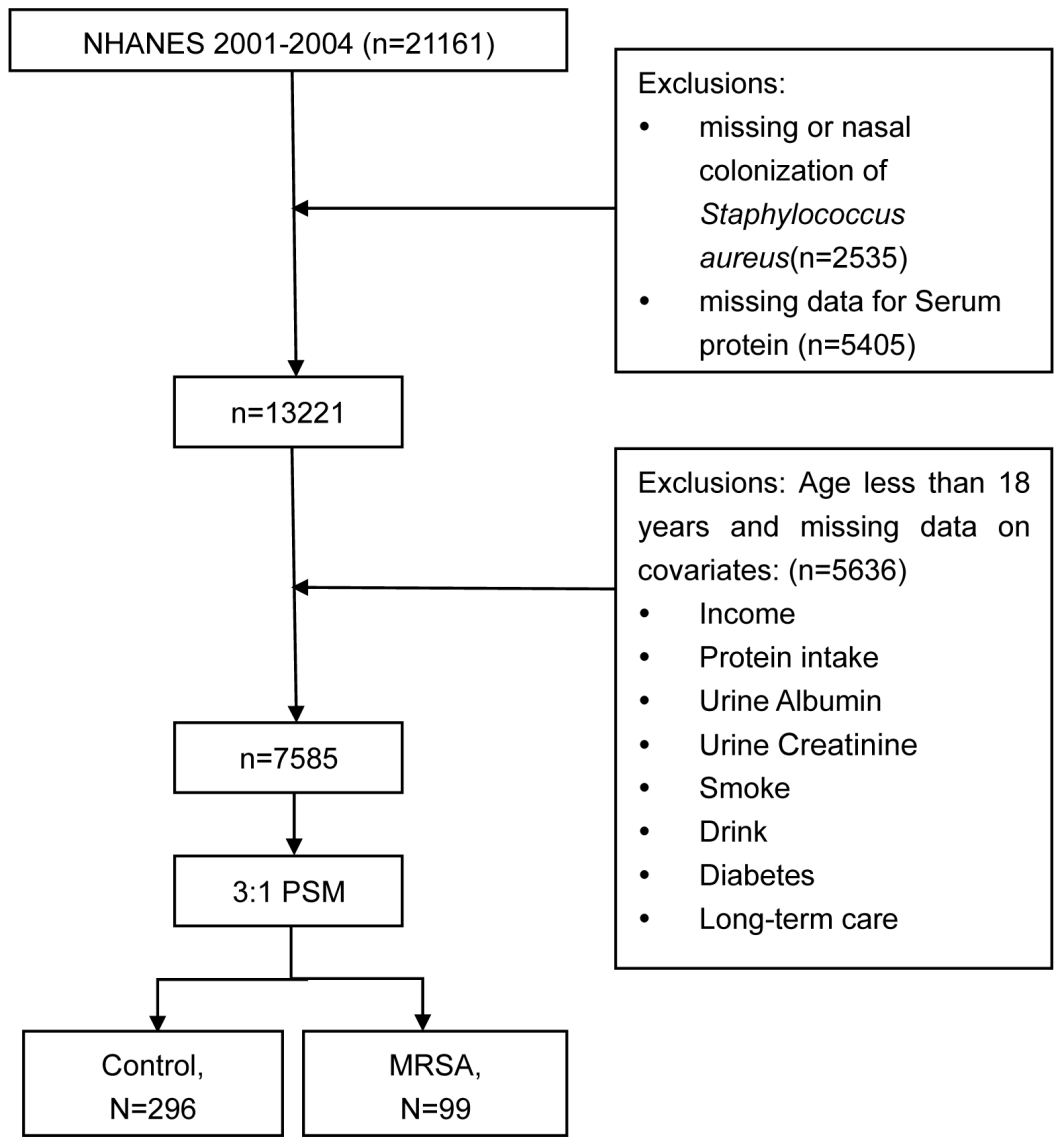
Flowchart of the study participants.

During the NHANES data cycle of 2001-2004, a total of 21,161 participants were enrolled. Initially, participants lacking data on nasal colonization of SA (n = 2535) were excluded. Subsequently, those with missing data on TP (n = 5405) were also excluded. Ultimately, after excluding missing values for covariates, a total of 7585 participants took part in this analysis. The sample selection process is shown in **Figure 1**.

### Assessment of TP, ALB, GLB and albumin-to-globulin ratio

The original biochemical parameters, including TP, ALB, and GLB, were obtained from the NHANES database. The measurements were performed in a standardized laboratory setting as follows: TP was quantified using a timed endpoint biuret method (LX20 analyzer, Beckman Coulter). In this reaction, alkaline copper ions form a colored chelate complex with peptide bonds in proteins, and the absorbance rate change at 545 nm is directly proportional to TP concentration. ALB was measured via a bichromatic digital endpoint assay (Bromcresol Purple, BCP method). At pH 5.2-6.8, BCP selectively binds to ALB, forming a complex with absorbance measured at 600 nm. This method minimizes nonspecific interactions with other serum proteins compared to traditional Bromcresol Green (BCG) assays. GLB levels were derived by subtracting ALB from TP. The AGR was calculated as ALB/GLB.

### Assessment of nasal colonization of MRSA

Nasal swab specimens were processed according to standardized protocols. Briefly, swabs were inoculated onto mannitol salt agar (MSA) and incubated at 35°C for 48 hours to isolate SA. Yellow colonies (mannitol fermenters) were subcultured onto blood agar plates (BAP) for purity confirmation. SA identification was performed using the Staphaurex latex agglutination kit (Thermo Fisher Scientific), with discrepancies resolved by tube coagulase testing (EDTA-rabbit plasma). Methicillin resistance was determined via oxacillin disk diffusion (1 μg, NCCLS criteria: ≤10 mm zone = resistant). Isolates resistant to oxacillin were classified as MRSA, while susceptible/intermediate isolates underwent additional broth microdilution MIC testing and molecular characterization (e.g., SCCmec typing) to confirm phenotypic resistance. MSSA classification required full susceptibility across both screening methods. Detailed laboratory procedures can be found on the NHANES website (21, 22).

### Covariates

Potential confounders initially included age, sex, race (Mexican American, non-Hispanic white, non-Hispanic black, and other races), income status, diabetes mellitus, daily protein intake, urinary protein level, smoking status, alcohol use, and whether the respondent had been admitted to a long-term care facility in the past 12 months. These factors were considered to be potentially associated with nasal MRSA colonization and serum proteins levels. A one-way logistic regression analysis was performed to screen for variables significantly associated with nasal MRSA colonization (P < 0.05) and the results are shown in **Supplementary Table 1**. The final confounders included age, gender, race (Mexican American, non-Hispanic white, non-Hispanic black, and other races), income status, diabetes mellitus, and whether or not the respondent had been admitted to a long-term care facility in the past 12 months. Income was dichotomized into annual income ≤ $45,000 and > $45,000. Diabetes was defined as a self-reported physician diagnosis based on the DIQ010 questionnaire. Use of long-term care services was defined as whether the respondent had been in a long-term care or rehabilitation facility in the past 12 months based on the HUQ082 questionnaire.

### Survey weights

Given the complexity of the NHANES survey design and to ensure unbiased national estimates (23), we followed the guidelines provided by the National Center for Health Statistics (NCHS) and used the sample weights WTMEC2YR, which represent the full sample 2-year MEC exam weights, to weight our analyses.

### Statistical analysis

Participants were divided into two groups based on nasal colonization of MRSA. Participants were divided into two groups based on nasal colonization of MRSA. As our study aimed to identify factors specific to MRSA (vs. non-MRSA colonization), MSSA cases were included in the reference group for statistical comparisons.

Continuous variables are reported as mean ± standard deviation (Mean ± SD), while categorical variables are expressed as N (%). Differences in baseline characteristics were assessed using Student’s t-tests and chi-square tests.

To balance the covariate differences between the MRSA-set and non-MRSA-set groups and ensure that they had similar covariate distributions, 1:3 propensity score matching (PSM) was performed. The association between TP, ALB, GLB, and AGR and nasal MRSA colonization was examined using logistic regression analysis, with results presented as odds ratios (OR) and 95% confidence intervals (95% CI). Initially, a crude model was analyzed with no covariate adjustment. Model 1 was adjusted for age, gender, race, and income. Model 2 included additional adjustments for long-term care use and diabetes. After converting TP and GLB from a continuous to a categorical variable, a trend test assessed the linear correlation between TP, GLB, and nasal MRSA colonization.

In addition, restricted cubic splines (RCS) were used to investigate potential non-linear associations between TP, GLB, and nasal MRSA colonization.

Subgroup analyses explored associations between different age groups, gender, race, income level, long-term care facility, and diabetes.

Additionally, we performed a number of sensitivity studies to evaluate the precision of our findings. We excluded outliers for TP and GLB and performed logistic regression analyses between TP, GLB, and MRSA colonization using data within the normal range.

A p-value of less than 0.05 was considered statistically significant. All analyses were performed using R version 4.4.2.

## Results

### Participant characteristic

**Table 1** summarizes the general characteristics of the study population. We ended up including 7585 participants in the analysis. The mean age of the participants was 46 ± 17 years, of which 3893 (51%) were female. Of the total number of participants, 99 (1.31%) had MRSA colonization. The mean TP level was 72.4 ± 4.7 g/L, the mean ALB level was 42.7 ± 3.3 g/L, the mean GLB level was 29.8 ± 4.3 g/L, and the mean AGR was 1.47± 0.26.

**Table 1:** Weight descriptive characteristics of participants with and without MRSA nasal colonization in the enrolled population of NHANES.

Variables	Unmatching				Matching^*^			
	Total(n=7585)	Control(n=7486)	MRSA^¶^(n=99)	P^‡^	Total(n=396)	Control(n=297)	MRSA^¶^(n=99)	P^‡^
Age, Mean±(SE)	46 (17)	46 (17)	53 (19)	**0.010**	53 (19)	54 (19)	53 (19)	0.653
TP (g/L), Mean±(SE)	72.4 (4.7)	72.5 (4.7)	70.3 (4.8)	**<0.001**	71.8 (5.0)	72.3 (4.9)	70.3 (4.8)	**0.003**
GLB (g/L), Mean±(SE)	29.8 (4.3)	29.8 (4.3)	28.7 (4.8)	**0.012**	30.0 (4.7)	30.4 (4.6)	28.7 (4.8)	**0.009**
ALB (g/L), Mean±(SE)	42.7 (3.3)	42.7 (3.3)	41.6 (3.7)	**0.009**	41.8 (3.3)	41.9 (3.2)	41.6 (3.7)	0.494
AGR, Mean±(SE)	1.47 (0.26)	1.46 (0.26)	1.49 (0.30)	0.295	1.43 (0.27)	1.41 (0.25)	1.49 (0.30)	**0.038**
Sex, n (%)				**0.004**				0.846
Male	3692 (49%)	3658 (49%)	34 (32%)		141 (35%)	107 (36%)	34 (34%)	
Female	3893 (51%)	3828 (51%)	65 (68%)		255 (65%)	190 (64%)	65 (66%)	
Race, n (%)				0.106				0.578
White	4166 (74%)	4104 (74%)	62 (79%)		261 (66%)	199 (67%)	62 (63%)	
Black	1343 (9.9%)	1324 (9.8%)	19 (13%)		75 (19%)	56 (18.9%)	19 (19%)	
Mexican American	1545 (7.2%)	1531 (7.2%)	14 (3.4%)		48 (11%)	34 (11.4%)	14 (14%)	
Other Race^5^	531 (8.9%)	527 (9.0%)	4 (5.0%)		12 (3.3%)	8 (2.7%)	4 (4.0%)	
Income, n (%)				**0.003**				0.966
<45000	4312 (53%)	4234 (54%)	78 (69%)		309 (79%)	231 (79%)	78 (79%)	
≥45000	3273 (47%)	3252 (46%)	21 (31%)		87 (21%)	66 (21%)	21 (21%)	
Diabetes, n (%)				**0.010**				0.414
No	6837 (90.1%)	6759 (90.3%)	78 (78.8%)		323 (82%)	245 (83%)	78 (79%)	
Yes	748 (9.9%)	727 (9.7%)	21 (21.2%)		73 (18%)	52 (17%)	21 (21%)	
Health facility in last 12month, n (%)				**0.003**				0.972
No	7511 (99%)	7419 (99%)	92 (97%)		366 (93%)	274 (93%)	92 (93%)	
Yes	74 (0.7%)	67 (0.7%)	7 (2.5%)		30 (7.1%)	23 (7.2%)	7 (7.1%)	

Mean ± SE for continuous variables: P value was calculated by weighted linear regression model. % for categorical variables: P value was calculated by weighted chi-square test. TP, Total protein; ALB, Albumin; GLB, Globulin; AGR, Albumin-to-Globulin Ratio; Total protein, albumin and globulin are all derived from standard biochemical tests. Other Race, Including Other Hispanic and Multi-Racial.

*: Propensity Matching Analysis.

‡: Kruskal-Wallis rank sum test; Pearson’s Chi-squared test.

¶: MRSA, Methicillin-resistant *Staphylococcus aureus*.

Values in boldface are significantly different (p < 0.05) from the reference group.

The relationship between nasal MRSA colonization and demographic characteristics was further analyzed by dividing the study population into 2 groups: the MRSA colonization group and the no MRSA colonization group. MRSA-colonized participants were older, had a higher proportion of females, lower incomes, and higher proportions of diabetics and long-term medical care recipients compared with those without MRSA implantation.

Baseline characteristics of the PSM-corrected population are shown in **Table 1**. After PSM correction, the distributions of the covariates were not statistically significant between the two groups, except for TP, GLB, and AGR, which were statistically different (p > 0.05) in the MRSA group (TP: p = 0.003; GLB: p = 0.009; AGR: p = 0.038; respectively, after matching).

### Associations of Serum proteins and nasal colonization of MRSA

**Table 2** demonstrates the results of the analyses of the relationship between nasal MRSA colonization and serum proteins in different univariate and multivariate regression models (crude model; model 1: adjusted for age, sex, race, income; model 2: adjusted for age, sex, race, income, diabetic patients, and long-term medical care recipients). After full adjustment of the models by the inclusion of covariates, nasal MRSA colonization was significantly and negatively associated with TP (OR = 0.92(95%CI 0.88, 0.96), indicating that for each unit increase in TP, the probability of nasal MRSA colonization decreased by 8%. Similarly, nasal MRSA colonization was significantly negatively correlated with GLB (OR = 0.91(95%CI 0.86, 0.97) and this relationship persisted after full adjustment of the model. However, the statistically significant difference between ALB and MRSA colonization disappeared after full adjustment of the model (P > 0.05), which may suggest that the negative correlation between TP and MRSA colonization may be mainly attributed to the role of GLB rather than ALB.

**Table 2:** Associations between TP, GLB, ALB, and AGR levels and MRSA nasal colonization.

Variable	Unmatched						Matching^*^					
	Crude model		Model 1		Model 2		Crude model		Model 1		Model 2	
	OR^¶^ (95% CI^¶^)	*p*	OR (95% CI)	*p*	OR (95% CI)	*p*	OR (95% CI)	*p*	OR (95% CI)	*p*	OR (95% CI)	*p*
TP (g/L)	0.9 (0.86, 0.94)	**<0.001**	0.92 (0.88, 0.96)	**<0.001**	0.92 (0.88, 0.96)	**<0.001**	0.92 (0.87, 0.97)	**0.003**	0.9 (0.85, 0.96)	**0.002**	0.9 (0.85, 0.96)	**0.002**
ALB (g/L)	0.91 (0.86, 0.97)	**0.004**	0.96 (0.89, 1.03)	0.245	1.01 (0.89, 1.04)	0.299	0.97 (0.88, 1.06)	0.492	-	-	-	-
GLB (g/L)	0.93 (0.88, 0.99)	**0.023**	0.91 (0.86, 0.97)	**0.007**	0.91 (0.86, 0.97)	**0.006**	0.92 (0.85, 0.98)	**0.016**	0.89 (0.82, 0.96)	**0.006**	0.89 (0.82, 0.96)	**0.006**
AGR	0.99 (0.68, 3.61)	0.287	-	-	-	-	3.21 (1.07, 9.63)	**0.038**	4.64 (1.42, 15.20)	**0.013**	4.62 (1.42, 15.08)	**0.014**

*: Propensity Matching Analysis.

¶: OR, Odds Ratio; CI, Confidence Interval.

TP, Total protein; ALB, Albumin; GLB, Globulin; AGR, Albumin-to-Globulin Ratio. Crude model was analyzed with no covariate adjustment. Model 1 was adjusted for age, gender, race, and income. Model 2 included additional adjustments for long-term care use and diabetes. Values in boldface are significantly different (p < 0.05) from the reference group.

Notably, only the negative correlation between TP, GLB and nasal MRSA colonization remained before and after matching (Matching: TP OR = 0.90(95%CI 0.85, 0.96); GLB OR = 0.89(95%CI 0.82, 0.96).

**Table 3** describes the decreased likelihood of nasal MRSA colonization with increasing quartiles of TP and GLB (TP Q4 vs. Q1: OR = 0.27(95%CI 0.14, 0.51); GLB Q4 vs. Q1: OR = 0.45(95%CI 0.27, 0.76)). In addition, the correlation between MRSA bacterial colonization along with TP and GLB persisted in model 1 (TP Q4 vs. Q1: OR = 0.31(95% CI 0.15, 0.63); GLB Q4 vs. Q1: OR = 0.37(95% CI 0.22, 0.63)) and model 2 (TP Q4 vs. Q1: OR = 0.32(95% CI 0.15, 0.66), P for trend = 0.011; GLB Q4 vs. Q1: OR = 0.34(95% CI, 0.19, 0.62), P for trend = 0.002). A significant correlation between GLB and nasal MRSA nasal colonization was still observed after propensity matching. (model 2: TP Q4 vs. Q1: OR = 0.21(95%CI 0.07, 0.59), P for trend = 0.018; GLB Q4 vs. Q1: OR = 0.28(95%CI 0.12, 0.67), P for trend = 0.005).

**Table 3:** Associations between TP, GLB, and MRSA colonization.

Variable	Unmatching			Matching^1^		
	Categories	OR^¶^ (95% CI^¶^)	P -value	Categories	OR (95% CI)	P -value
TP Crude model	Q1	Ref	Ref	Q1	Ref	Ref
	Q2	0.39 (0.21, 0.73)	**0.004**	Q2	0.36 (0.19, 0.71)	**0.005**
	Q3	0.51 (0.26, 1.00)	0.051	Q3	0.60 (0.26, 1.43)	0.239
	Q4	0.27 (0.14, 0.51)	**<0.001**	Q4	0.26 (0.11, 0.60)	**0.003**
	P for trend	–	**0.001**	P for trend	–	**0.012**
TP Model 1	Q1	Ref	Ref	Q1	Ref	Ref
	Q2	0.45 (0.25, 0.81)	**0.010**	Q2	0.35 (0.18, 0.69)	**0.004**
	Q3	0.60 (0.30, 1.19)	0.135	Q3	0.58 (0.25, 1.35)	0.196
	Q4	0.31 (0.15, 0.63)	**0.002**	Q4	0.24 (0.09, 0.64)	**0.006**
	P for trend	–	**0.010**	P for trend	–	**0.018**
TP Model 2	Q1	Ref	Ref	Q1	Ref	Ref
	Q2	0.45 (0.25, 0.83)	**0.013**	Q2	0.33 (0.17, 0.64)	**0.003**
	Q3	0.62 (0.31, 1.24)	0.162	Q3	0.55 (0.23, 1.31)	0.166
	Q4	0.32 (0.15, 0.66)	**0.004**	Q4	0.21 (0.07, 0.59)	**0.005**
	P for trend	–	**0.011**	P for trend	–	**0.018**
Variable	Unmatching			Matching		
	Categories	OR (95% CI)	P -value	Categories	OR (95% CI)	P -value
GLB Crude model	Q1	Ref	Ref	Q1	Ref	Ref
	Q2	0.67 (0.39, 1.16)	0.144	Q2	0.79 (0.37, 1.71)	0.537
	Q3	0.52 (0.32, 0.84)	**0.010**	Q3	0.49 (0.25, 0.95)	**0.037**
	Q4	0.45 (0.27, 0.76)	**0.005**	Q4	0.37 (0.19, 0.75)	**0.007**
	P for trend	–	**0.004**	P for trend	–	**0.003**
GLB Model 1	Q1	Ref	Ref	Q1	Ref	Ref
	Q2	0.67 (0.39, 1.12)	0.120	Q2	0.78 (0.38, 1.62)	0.494
	Q3	0.46 (0.28, 0.77)	**0.004**	Q3	0.45 (0.22, 0.91)	**0.028**
	Q4	0.37 (0.22, 0.63)	**0.001**	Q4	0.33 (0.15, 0.72)	**0.008**
	P for trend	–	**0.002**	P for trend	–	**0.005**
GLB Model 2	Q1	Ref	Ref	Q1	Ref	Ref
	Q2	0.68 (0.40, 1.16)	0.145	Q2	0.79 (0.38, 1.64)	0.504
	Q3	0.46 (0.26, 0.80)	**0.008**	Q3	0.42 (0.20, 0.86)	**0.020**
	Q4	0.34 (0.19, 0.62)	**0.001**	Q4	0.28 (0.12, 0.67)	**0.007**
	P for trend	–	**0.002**	P for trend	–	**0.005**

*: Propensity Matching Analysis.

¶: OR, Odds Ratio; CI, Confidence Interval.

TP, Total protein; GLB, Globulin. Crude model was analyzed with no covariate adjustment. Model 1 was adjusted for age, gender, race, and income. Model 2 included additional adjustments for long-term care use and diabetes. Values in boldface are significantly different (p < 0.05) from the reference group.

**Figure 2:**
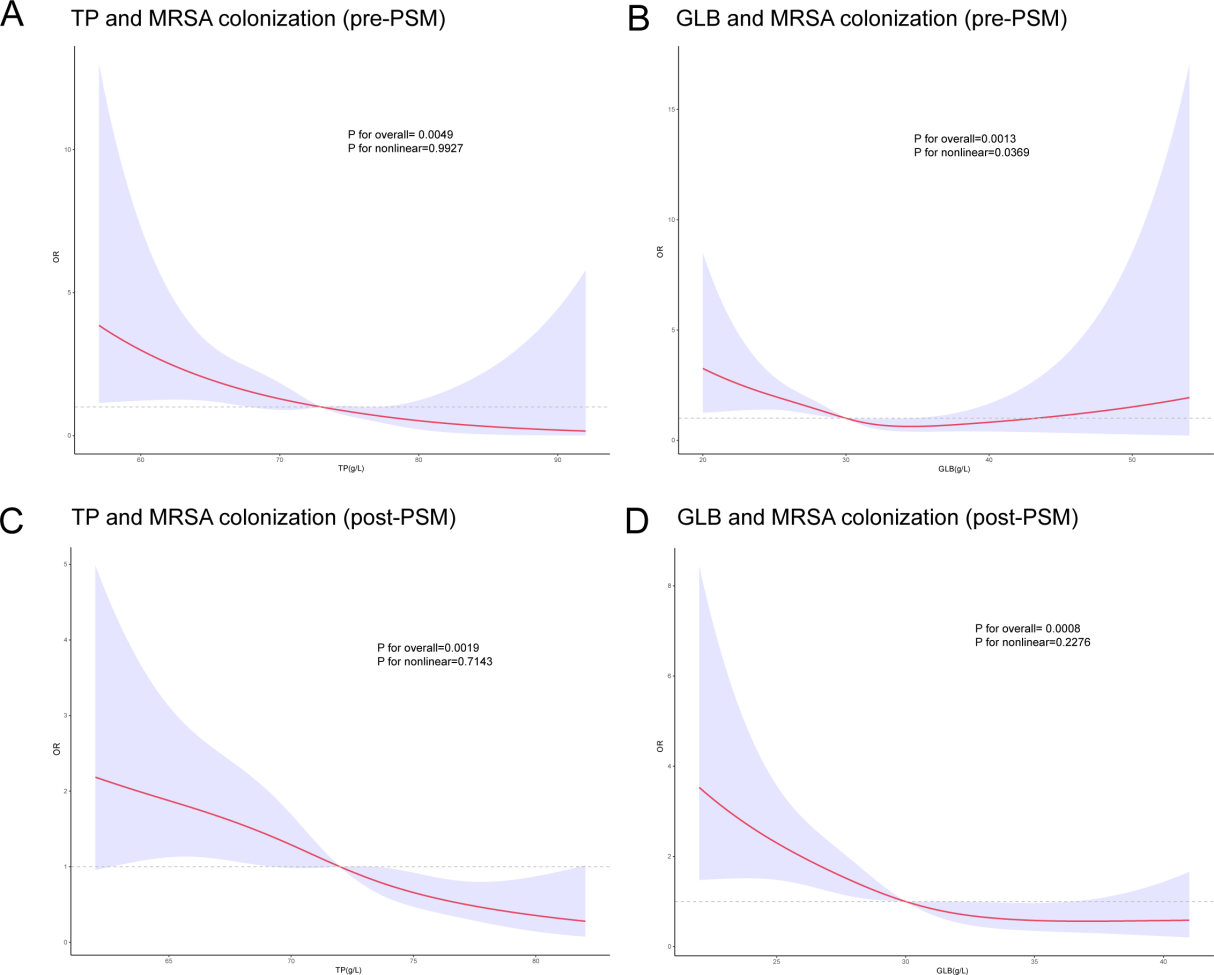
Restricted cubic spline (RCS) plots of total protein (TP), globulin (GLB), and methicillin-resistant Staphylococcus aureus (MRSA) nasal colonization before and after propensity score matching (PSM). Panels **(A, B)** illustrate the RCS plots of TP and GLB in relation to MRSA colonization before PSM; panels **(C, D)** show the corresponding analyses after PSM.

As shown in **Figure 2**, the smoothed curve-fitting analysis corrected for confounders further confirmed that MRSA fixation was significantly negatively correlated with TP and GLB levels (P<0.05). Notably, the negative correlation between MRSA colonization and TP and GLB remained statistically significant (P<0.05) even after PSM.

### Subgroup analysis

**Figure 3:**
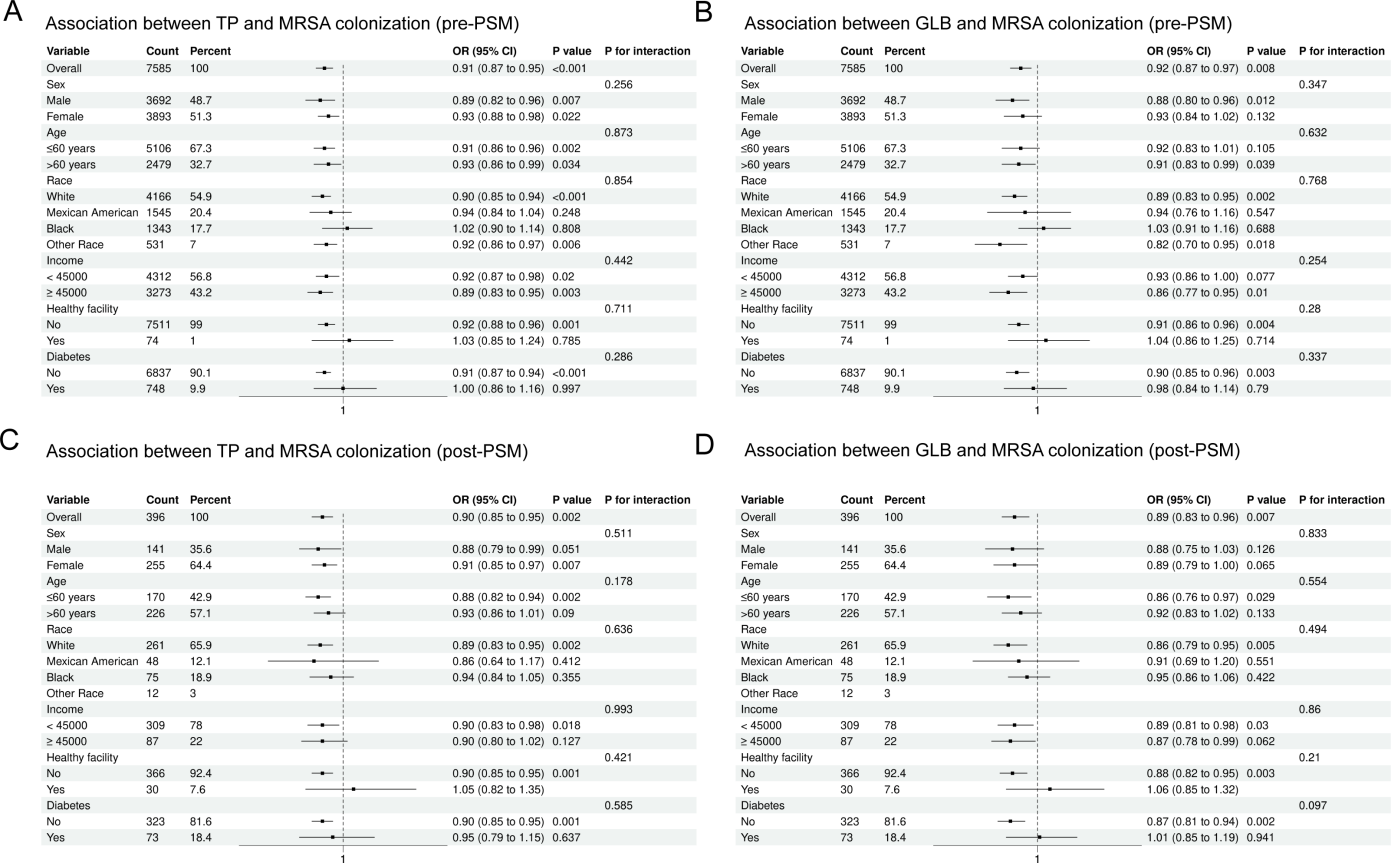
Subgroup analysis stratified by demographic and clinical variables before and after propensity score matching (PSM). **(A)** Total protein (TP) and MRSA colonization, pre-PSM; **(B)** Globulin (GLB) and MRSA colonization, pre-PSM; **(C)** TP and MRSA colonization, post-PSM; **(D)** GLB and MRSA colonization, post-PSM.

In this study, we used subgroup analyses to verify whether the relationship between TP, GLB, and MRSA nasal colonization was affected by age, gender, race, income, diabetes mellitus, and structure of long-term medical care received. **Figure 3** shows the results of the subgroup analyses for serum proteins and MRSA colonization. After adjusting for confounders, we found that the negative correlation between TP, GLB, and MRSA nasal colonization before and after PSM was generally significant in all subgroups. We found no interaction between the results of the subgroup analyses between all populations (P > 0.05 for interaction).

### Sensitivity analysis

In order to verify the real reliability of the results, we took the following approaches for sensitivity analysis: We excluded the outliers of TP and GLB and used data within the normal range for TP and GLB and MRSA colonization logistic regression analyses. The results of the sensitivity analyses were consistent with our results above (see **Supplementary Table 2**).

## Discussion

In this study, we collected data related to TP, GLB, and ALB from the NHANES 2001–2004 dataset and performed observational correlation analyses of their association with MRSA nasal colonization. We found a significant negative correlation between MRSA nasal colonization and TP and GLB. After adjusting for potential confounders, higher levels of TP and GLB were still associated with lower rates of MRSA nasal colonization. This correlation was evident even when TP and GLB levels were analyzed in quartiles. We further confirmed the negative correlation between TP and GLB and MRSA nasal colonization rates using RCS. Sensitivity analyses and subgroup analyses further demonstrated the robustness of the results. To the best of our knowledge, this study represents the first to utilize data from the NHANES to demonstrate an association between MRSA nasal colonization and TP and GLB. These findings provide new insights into the relationship between TP, GLB, and MRSA nasal colonization, suggesting that TP and GLB may be potentially informative in the study and clinical evaluation of MRSA colonization.

Several studies have found reduced TP and ALB levels in patients with MRSA colonization versus patients with MRSA infection, which may reflect the fact that malnourished patients are more likely to acquire MRSA colonization (12–17). However, these reported cases were limited to the elderly population as well as to the dialysis population, and only changes in ALB, not GLB, were seen. In addition, it has been found that there are differences in adaptive humoral immune responses between SA carriers and non-carriers. Carriers frequently report higher serum immunoglobulin G (IgG) titers compared with noncarriers (24–27). Also, higher median IgA levels of several staphylococcal proteins have been noted in persistent carriers (28). However, one study also found no association between SA antigens and the humoral immune response (29). It is worth noting that the above studies mainly tested specific IgG/IgA antibodies against SA, so even if the IgG levels of specific antigens were elevated, this would not necessarily lead to significant changes in the overall levels of GLB. Serum GLB, also known as the gamma gap or protein gap, is usually calculated as the difference between TP and ALB, and therefore includes all non-albumin proteins including globulins, fibrinogen, C-reactive proteins, interleukins, leukotrienes, and other regulatory and pro-thrombotic proteins (30). Serum GLB values may be elevated in conditions such as infections, inflammation, and liver and connective tissue diseases, whereas lower serum GLB values may be caused by malnutrition and nephrotic syndrome, due to reduced renal synthesis and protein loss, respectively (31, 32). Furthermore, low levels of immunoglobulin may be indicative of low immune function, and malnutrition and low immune function lead to increased susceptibility to infections (33–35). This may help to explain why MRSA colonization is more likely in people with low GLB. MRSA colonization is more common in older people, which may be due to the gradual decline in immune function with age, which leads to a higher likelihood of colonization (36). In addition, patients such as hemopoietic stem cell transplant (HSCT) recipients or those on long-term immunosuppressive therapy are at a 2- to 3-fold increased risk of MRSA and other MDRO colonization. Among colonized patients, 30% develop active infections, including carbapenem-resistant gram-negative bacteria (CR-GNB) (14%) and VRE (8%) (11, 37, 38), a consequence of impaired immune function. Notably, while the overall burden of MDRO infections in immunocompromised patients in the intensive care unit (ICU) remains low, MRSA colonization rates persist at high levels, underscoring its unique adaptability and resilience in healthcare settings (18).

Since 2005, simultaneous decreases in MRSA infections have been demonstrated in multiple populations in the United States and Europe (39–43), particularly in blood and soft tissue infections, which may be attributed to the awareness and implementation of local and worldwide infection prevention measures in many healthcare settings. However, even with advances in antibiotics, active surveillance efforts, and infection prevention, MRSA remains a major pathogen with consistently high mortality rates (8). MRSA colonization increases the risk of infection, and how to correctly and rapidly identify MRSA colonization remains a critical issue, yet current culture - based detection methods, while clinically practical, lack the sensitivity of molecular assays.

Our study offers several significant strengths and implications. Notably, it is based on NHANES and uses a weighted design, which guarantees an adequate sample size, enhances credibility, and ensures strong representation. Second, by using PSM, we effectively reduced selection bias and increased the confidence in our findings, thereby eliminating the confounding factors that often occur in observational studies. Third, by using RCS analyses, we again demonstrated the near-linear negative correlation association between TP, GLB, and MRSA nasal colonization. In addition, we performed subgroup analyses to further explore the relationship between TP, GLB, and MRSA nasal colonization in different populations.

However, this study still has some limitations. Firstly, given our cross-sectional design, we were unable to make causal inferences. Secondly, the NHANES data on MRSA colonization only exists for the years 2001-2004, which is a long time ago and may have biased the results of the study. In addition, the presence of unidentified confounders may still have an impact, thus limiting the interpretation of the results of our study. Therefore, further prospective clinical studies are needed to elucidate the complex relationship between TP, GLB, and MRSA nasal colonization.

## Conclusion

In this cross-sectional study, there was a significant negative correlation between TP, GLB and nasal MRSA colonization. However, due to the nature of the design of this study, causal inferences could not be made, and further prospective studies are needed to validate these findings and investigate the causal relationship between TP, GLB, and nasal MRSA colonization, and the specific mechanisms underlying the role of these biomarkers in MRSA colonization and their clinical implications require further exploration.

## Data Availability

The original contributions presented in the study are included in the article/**Supplementary Material**. Further inquiries can be directed to the corresponding authors.

## References

[1] Wertheim HF Melles DC Vos MC van Leeuwen W van Belkum A Verbrugh HA. The role of nasal carriage in *Staphylococcus aureus* infections. Lancet Infect Dis. (2005) 5:751–62. doi: 10.1016/S1473-3099(05)70295-4 16310147

[2] Gould D. *Staphylococcus aureus*: a review of the literature. J Clin Nurs. (1995) 4(1):5–12. doi: 10.1111/j.1365-2702.1995.tb00004.x 7704377

[3] Wolk DM Struelens MJ Pancholi P Davis T Della-Latta P Fuller D. Rapid Detection of *Staphylococcus aureus* and Methicillin-Resistant S. aureus (MRSA) in Wound Specimens and Blood Cultures: Multicenter Preclinical Evaluation of the Cepheid Xpert MRSA/SA Skin and Soft Tissue and Blood Culture Assays. J Clin Microbiol. (2009) 47:823–6. doi: 10.1128/JCM.01884-08 PMC2650929 19144803

[4] Whitby M McLaws ML Berry G. Risk of death from methicillin-resistant *Staphylococcus aureus* bacteraemia: a meta-analysis. Med J Aust. (2001) 175:264–7. doi: 10.5694/j.1326-5377.2001.tb143562.x 11587259

[5] Fortuin-de Smidt MC Singh-Moodley A Badat R Quan V Kularatne R Nana T. *Staphylococcus aureus* bacteraemia in Gauteng academic hospitals, South Africa. Int J Infect Dis. (2015) 30:41–8. doi: 10.1016/j.ijid.2014.10.011 25448331

[6] Antonanzas F Lozano C Torres C. Economic features of antibiotic resistance: the case of methicillin-resistant *staphylococcus aureus* . PharmacoEconomics. 33(4):285–325. doi: 10.1007/s40273-014-0242-y 25447195

[7] Thampi N Showler A Burry L Bai AD Steinberg M Ricciuto DR. Multicenter study of health care cost of patients admitted to hospital with *Staphylococcus aureus* bacteremia: Impact of length of stay and intensity of care. Am J Infect Control. (2015) 43:739–44. doi: 10.1016/j.ajic.2015.01.031 25769617

[8] Collaborators G 2021 AR. Global burden of bacterial antimicrobial resistance 1990–2021: a systematic analysis with forecasts to 2050. Lancet Lond Engl. (2024) 404:1199–226. doi: 10.1016/S0140-6736(24)01867-1 PMC11718157 39299261

[9] Kourtis AP. Vital signs: epidemiology and recent trends in methicillin-resistant and in methicillin-susceptible *staphylococcus aureus* bloodstream infections — United states. MMWR Morb Mortal Wkly Rep. (2019) 68(9):214–9. doi: 10.15585/mmwr.mm6809e1 PMC6421967 30845118

[10] CDC. Antibiotic resistance threats in the United States, 2013 . Available online at: https://stacks.cdc.gov/view/cdc/20705 (Accessed March 15, 2024).

[11] Bruyneel A Miesse I Mathieu D Djuidjé Yuemo C Simon A. Prevalence and factors associated with methicillin-resistant *Staphylococcus aureus* colonization on admission to geriatric care units: impact on screening practices. J Hosp Infect. (2024) 146:109–15. doi: 10.1016/j.jhin.2024.01.014 38309666

[12] Honjo N Washio M Ito Y Fujishima M. Isolation of methicillin-resistant *Staphylococcus aureus* on admission to a geriatric hospital. Nihon Ronen Igakkai Zasshi Jpn J Geriatr. (1997) 34:147–50. doi: 10.3143/geriatrics.34.147 9125891

[13] Takeda S Tatara I Kono K Arakawa K. Relation between nutrition of patients and methicillin-resistant *Staphylococcus aureus* (MRSA). Kansenshogaku Zasshi. (1996) 70:354–9. doi: 10.11150/kansenshogakuzasshi1970.70.354 8690950

[14] Yeoh LY. Methicillin-resistant *Staphylococcus aureus* carriage in hospitalized chronic hemodialysis patients and its predisposing factors. Hemodial Int Int Symp Home Hemodial. (2014) 18(1):142–7. doi: 10.1111/hdi.12061 23763574

[15] Lai CF Liao CH Pai MF Chu FY Hsu SP Chen HY. Nasal carriage of methicillin-resistant *Staphylococcus aureus* is associated with higher all-cause mortality in hemodialysis patients. Clin J Am Soc Nephrol CJASN. (2011) 6:167–74. doi: 10.2215/CJN.06270710 PMC3022239 20947786

[16] Hadley AC Karchmer TB Russell GB McBride DG Freedman BI. The prevalence of resistant bacterial colonization in chronic hemodialysis patients. Am J Nephrol. (2007) 27:352–9. doi: 10.1159/000103383 17541264

[17] Aizen E Ljubuncic Z Ljubuncic P Aizen I Potasman I. Risk factors for methicillin-resistant *Staphylococcus aureus* colonization in a geriatric rehabilitation hospital. J Gerontol A Biol Sci Med Sci. (2007) 62:1152–6. doi: 10.1093/gerona/62.10.1152 17921430

[18] Mangalea MR Halpin AL Haile M Elkins CA McDonald LC. Decolonization and pathogen reduction approaches to prevent antimicrobial resistance and healthcare-associated infections. Emerging Infect Dis. 30(6):1069–76. doi: 10.3201/eid3006.231338 PMC11138981 38781679

[19] Juul F Parekh N Martinez-Steele E Monteiro CA Chang VW. Ultra-processed food consumption among US adults from 2001 to 2018. Am J Clin Nutr. (2022) 115:211–21. doi: 10.1093/ajcn/nqab305 34647997

[20] Chen F Du M Blumberg JB Chui KKH Ruan M Rogers G. Association between dietary supplement use, nutrient intake, and mortality among US adults: A cohort study. Ann Intern Med. (2019) 170:604–13. doi: 10.7326/M18-2478 PMC6736694 30959527

[21] NHANES 2001–2002 laboratory methods . Available online at: https://wwwn.cdc.gov/nchs/nhanes/continuousnhanes/labmethods.aspx?BeginYear=2001 (Accessed March 15, 2024).

[22] NHANES 2003–2004 laboratory methods . Available online at: https://wwwn.cdc.gov/nchs/nhanes/continuousnhanes/labmethods.aspx?BeginYear=2003 (Accessed March 15, 2024).

[23] CDC. National health and nutrition examination survey (2024). Available online at: https://www.cdc.gov/nchs/nhanes/index.html (Accessed March 15, 2024).

[24] Swierstra J Debets S de Vogel C Lemmens-den Toom N Verkaik N Ramdani-Bouguessa N. IgG4 subclass-specific responses to *Staphylococcus aureus* antigens shed new light on host-pathogen interaction. Infect Immun. (2015) 83(2):492–501. doi: 10.1128/IAI.02286-14 PMC4294233 25404029

[25] Colque-Navarro P Jacobsson G Andersson R Flock JI Möllby R. Levels of antibody against 11 *Staphylococcus aureus* antigens in a healthy population. Clin Vaccine Immunol CVI. (2010) 17:1117–23. doi: 10.1128/CVI.00506-09 PMC2897265 20445005

[26] van Belkum A Verkaik NJ de Vogel CP Boelens HA Verveer J Nouwen JL. Reclassification of staphylococcus aureus nasal carriage types. J Infect Dis. (2009) 199(12):1820–6. doi: 10.1086/599119 19419332

[27] Ghasemzadeh-Moghaddam H van Wamel W van Belkum A Hamat RA Neela VK. Differences in humoral immune response between patients with or without nasal carriage of *Staphylococcus aureus* . Eur J Clin Microbiol Infect Dis. (2017) 36:451–8. doi: 10.1007/s10096-016-2817-3 27815779

[28] Verkaik NJ de Vogel CP Boelens HA Grumann D Hoogenboezem T Vink C. Anti-staphylococcal humoral immune response in persistent nasal carriers and noncarriers of Staphylococcus aureus. J Infect Dis. (2009) 199:625–32. doi: 10.1086/596743 19199541

[29] Espinosa-Gongora C Dahl J Elvstrøm A van Wamel WJ Guardabassi L. Individual predisposition to *staphylococcus aureus* colonization in pigs on the basis of quantification, carriage dynamics, and serological profiles. Appl Environ Microbiol. (2015) 81:1251–6. doi: 10.1128/AEM.03392-14 PMC4309706 25501475

[30] Sun L Chen T Zhu Y Zhou W Li P. Albumin-globulin ratio and mortality in patients on peritoneal dialysis: a retrospective study. BMC Nephrol. (2020) 21(1):51. doi: 10.1186/s12882-020-1707-1 32059708 PMC7023751

[31] Walker HK Hall WD Hurst JW eds. Clinical methods: the history, physical, and laboratory examinations. 3rd ed. Boston: Butterworths (1990). Available online at: http://www.ncbi.nlm.nih.gov/books/NBK201/. 21250045

[32] Retrospective cohort study of 148 patients with polyclonal gammopathy. 10.4065/76.5.476 11357794

[33] Kostinova AM Akhmatova NK Latysheva EA Dagil YA Klimova SV Vlasenko AE. Assessment of immunogenicity of adjuvanted quadrivalent inactivated influenza vaccine in healthy people and patients with common variable immune deficiency. Front Immunol. (2020) 11:1876. doi: 10.3389/fimmu.2020.01876 32973775 PMC7466564

[34] Liese JG Jendrossek V Jansson A Petropoulou T Kloos S Gahr M. Chronic granulomatous disease in adults. Lancet Lond Engl. (1996) 347:220–3. doi: 10.1016/S0140-6736(96)90403-1 8551880

[35] Peacock SJ Justice A Griffiths D de Silva GDI Kantzanou MN Crook D. Determinants of acquisition and carriage of *staphylococcus aureus* in infancy. J Clin Microbiol. (2003) 41(12):5718–25. doi: 10.1128/JCM.41.12.5718-5725.2003 PMC308978 14662966

[36] Graham PL Lin SX Larson EL. A U.S. population-based survey of *Staphylococcus aureus* colonization. Ann Intern Med. (2006) 144(5):318–25. doi: 10.7326/0003-4819-144-5-200603070-00006 16520472

[37] Wang J Wang M Huang Y Zhu M Wang Y Zhuo J. Colonization pressure adjusted by degree of environmental contamination: A better indicator for predicting methicillin-resistant *Staphylococcus aureus* acquisition. Am J Infect Control. (2011) 39:763–9. doi: 10.1016/j.ajic.2010.11.012 21600671

[38] Almohaya A Fersovich J Weyant RB Fernández García OA Campbell SM Doucette K. The impact of colonization by multidrug resistant bacteria on graft survival, risk of infection, and mortality in recipients of solid organ transplant: systematic review and meta-analysis. Clin Microbiol Infect. (2024) 30:1228–43. doi: 10.1016/j.cmi.2024.03.036 38608872

[39] Landrum ML Neumann C Cook C Chukwuma U Ellis MW Hospenthal DR. Epidemiology of *Staphylococcus aureus* blood and skin and soft tissue infections in the US military health system, 2005-2010. JAMA. (2012) 308(1):50–9. doi: 10.1001/jama.2012.7139 22760291

[40] Kallen AJ Mu Y Bulens S Reingold A Petit S Gershman K. Health care-associated invasive MRSA infections, 2005-2008. JAMA. (2010) 304(6):641–8. doi: 10.1001/jama.2010.1115 20699455

[41] Dantes R Mu Y Belflower R Aragon D Dumyati G Harrison LH. National burden of invasive methicillin-resistant *staphylococcus aureus* infections, United States, 2011. JAMA Intern Med. (2013) 173:1970–8. doi: 10.1001/jamainternmed.2013.10423 PMC10887428 24043270

[42] de Kraker MEA Jarlier V Monen JCM Heuer OE van de Sande N Grundmann H. The changing epidemiology of bacteraemias in Europe: trends from the European Antimicrobial Resistance Surveillance System. Clin Microbiol Infect Off Publ Eur Soc Clin Microbiol Infect Dis. (2013) 19:860–8. doi: 10.1111/1469-0691.12028 23039210

[43] Klein EY Mojica N Jiang W Cosgrove SE Septimus E Morgan DJ. Trends in methicillin-resistant *staphylococcus aureus* hospitalizations in the United States, 2010-2014. Clin Infect Dis Off Publ Infect Dis Soc Am. (2017) 65(11):1921–3. doi: 10.1093/cid/cix640 29020322

