# Supplementary material for: Associations between total protein, globulin, and nasal Methicillin-Resistant Staphylococcus aureus (MRSA) colonization in US adults: results from the national health and nutrition examination survey 2001–2004
Source: Front Immunol. 2025 May 30;16:1585718. doi: 10.3389/fimmu.2025.1585718 (PMC12162468; doi:10.3389/fimmu.2025.1585718)
Supplement: Supplementary file 1 [file Table1.docx]

Supplementary Table 1: Weighted Univariate Logistic Regression Analysis for Variables Associated with Nasal Methicillin-resistant *Staphylococcus aureus* (MRSA) Colonization

| **Variable** | **OR (95% CI)** | **P -value** |
| --- | --- | --- |
| **TP (g/L)** | 0.90 (0.86, 0.94) | **<0.001** |
| **GLB (g/L)** | 0.93 (0.88, 0.99) | **0.025** |
| **ALB (g/L)** | 0.91 (0.86, 0.97) | **0.006** |
| AGR | 1.55 (0.70, 3.39) | 0.278 |
| **Age** | 3.07 (1.97, 4.78) | **<0.001** |
| **Sex** | 2.07 (1.29, 3.30) | **0.006** |
| **Income** | 0.38 (0.21, 0.70) | **0.004** |
| **Race** | 0.44 (0.23, 0.84) | **0.020** |
| Drinking | 1.14 (0.65, 2.01) | 0.633 |
| Smoke | 1.59 (1.01, 2.51) | 0.057 |
| **Health facility in last 12month** | 3.97 (1.62, 9.71) | **0.005** |
| **Diabetes** | 2.19 (1.24, 3.87) | **0.011** |
| Protein intake (gm) | 1.00 (0.99, 1.00) | 0.429 |
| Urine Albumin (mg/L) | 1.00 (1.00, 1.00) | 0.074 |
| Urine Creatinine (mg/L) | 1.00 (1.00, 1.00) | 0.991 |

TP—Total protein; GLB—Globulin; ALB—Albumin; AGR—Albumin-to-Globulin Ratio; Values in boldface are significantly different (p < 0.05) from the reference group.
