# Supplementary material for: Associations between total protein, globulin, and nasal Methicillin-Resistant Staphylococcus aureus (MRSA) colonization in US adults: results from the national health and nutrition examination survey 2001–2004
Source: Front Immunol. 2025 May 30;16:1585718. doi: 10.3389/fimmu.2025.1585718 (PMC12162468; doi:10.3389/fimmu.2025.1585718)
Supplement: Supplementary file 2 [file Table2.docx]

Supplementary Table 2: Results of the sensitivity analyses

| **Variable** | **Crude model** | | **Model 1** | | **Model 2** | |
| --- | --- | --- | --- | --- | --- | --- |
|  | **OR^1^**  **(95% CI^1^)** | ***p*** | **OR**  **(95% CI)** | ***p*** | **OR**  **(95% CI)** | ***p*** |
| **TP^2^ (g/L)** | 0.90(0.87,0.93) | **<0.001** | 0.92(0.88, 0.95) | **<0.001** | 0.92(0.88,0.95) | **<0.001** |
| **GLB^2^ (g/L)** | 0.92(0.87,0.98) | **<0.001** | 0.91(0.85,0.96) | **0.003** | 0.90(0.85,0.96) | **0.002** |

1: OR = Odds Ratio, CI = Confidence Interval.

2: TP—Total protein; ALB—Albumin; GLB—Globulin; AGR—Albumin-to-Globulin Ratio.

Crude model was analyzed with no covariate adjustment. Model 1 was adjusted for age, gender, race, and income. Model 2 included additional adjustments for long-term care use and diabetes. Values in boldface are significantly different (p < 0.05) from the reference group.
